# Supplementary material for: Cryptic diploid lineage of Betula ermanii at its southern boundary populations in Japan
Source: PLoS One. 2024 Jul 18;19(7):e0307023. doi: 10.1371/journal.pone.0307023 (PMC11257223; doi:10.1371/journal.pone.0307023)
Supplement: Attachment — Submitted filename: Response to Reviewers.docx [file pone.0307023.s005.docx]

**Responds to the comments by Dr. Maidelyn R. Peregrin from at May 8, 2024**

1. Thank you for providing the necessary copyright information for the map image in Figure 1. However, before we proceed, we kindly ask that you please respond to the following queries regarding Figures 2, 3, 4, and 6:

1) We have noted that there are additional images included in Figures 2, 3, 4, and 6 in your submission. At this time, please explain where the authors obtained the images in Figures 2, 3, 4, and 6 or if the authors created these images themselves.

We have already answered the above question as follow of the response of April 18, 2024.

The images in Figures 2, 3, 4 and 6 were created by ourseslves. Then, we have added such explanations in the captions of Figures 2, 3, 4 and 6.

2) Please also state if any of the images in Figures 2, 3, 4, and 6 have been previously published or copyrighted.

There is not previously published or copyrighted in Figures 2, 3, 4, and 6.

**Responds to the comments by Dr. Maidelyn R. Peregrin from at April 18, 2024**

1) We have re-created the Figure 1 using the shapefiles of land boundary from Natural Earth (<https://www.naturalearthdata.com/>) instead of “TM World Borders Dataset 0.3”. Actually, the files from The Geospatial Information Authority of Japan can be used under CC BY 4.0 (<https://www.gsi.go.jp/kikakuchousei/kikakuchousei40182.html>). Unfortunately, this page was written only in Japanese. Then, we uploaded the image of this part translated in English as an other file.

1a) The map image in Figure 1 was made in the open access article <https://doi.org/10.1038/s41437-023-00655-0>. Those articles are published under CC BY 4.0 (<https://www.nature.com/hdy/open-access> ). Then, we did not need any permission for using this in Fig 1.

1b) We are not applicable for this issue.

1c) We have re-created the Figure 1 using the shapefiles of continental boundary from Natural Earth (<https://www.naturalearthdata.com/>) instead of “TM World Borders Dataset 0.3”.

2) The images in Figures 2, 3, 4 and 6 were created by ourselves. Then, we have added such explanations in the captions of Figures 2, 3, 4 and 6.

**Responds to Academic Editor**:

1. Our revised manuscript was made according to PLOS ONE's style requirements.

2. We have added the ethics statement and the full name of the authority that approved our sample collection in Methods section.

3. When we submit, we will provide the correct grant numbers for awards we received for this study in ‘Funding Information’.

4. The funders had no role in study design, data collection and analysis, decision to publish, or preparation of the manuscript.

5. We have provided an ORCID iD for the corresponding author.

6. We have added the ethics statement and the full name of the authority that approved our sample collection in Methods section in L 150-156.

7. The shapefiles of land boundary from Natural Earth (<https://www.naturalearthdata.com/>) The base map was made using files from The Geospatial Information Authority of Japan (<https://www.gsi.go.jp/kiban/index.html>). We have added this description in the caption of Fig 1. These files allow unrestricted use and distribution under the CC BY 4.0 licence. Then, we did not need any permission for using those in Fig 1.

**Responds to Reviewer #1**:

We greatly appreciate your extensive time and effort in providing the meaningful suggestions needed to publish these findings. As your comments, number of samplings for ploidy estimation except southern boundary populations is very small (only two individuals for each population). Based on the RAD-seq analysis (Aihara et al. 2023), it was observed that SHK, one of the southern boundary populations, could potentially be diploid. To confirm this observation, we conducted flow cytometry. Consequently, with the exception of three southern boundary populations, we sampled two samples from each population as controls. We have added such description in Materials L124-127. Finding of cryptic diploid individuals in *B. ermanii* is first report, and we believe that our manuscript has great impact and many readers show much attention.

Line 78: Authors have discussed Betula species, but not clear which species authors are talking about.

**REPLY**: Thank you for the comment. We revised the wording ‘related species’ to ‘separate species’ to clarify meaning in L80.

Line 80: How same species as been named differently in different regions? What is the acceptable botanical name of the species?

**REPLY**: Thank you for the comment. As many plant taxa, *B. ermanii* named differently in different regions by local botanist and researcher, and now combined as one species. We have added acceptable Japanese botanical name of the species in L80.

Line 87: Authors are talking about which species?

**REPLY**: We have added species name in L90.

Line 285: Authors stated that B. costata is diploid progenitor of B. ermanii. But, in next lines 286-87, authors are confirming that both are same species. And later on, authors also mentioning inadequate study related to this.

**REPLY**: Thank you for pointing it out. *Betula ermanii* and *B. costata* is separate species. We have revised sentences to clarify what we mean in L306-308.

Line 295-297: Authors agreed to the morphological similarity of their diploid specimen to the B. shikokiana, which is confusing, weather both are similar species or different.

**REPLY**: Thank you for the comment. Now *B. shikokiana* combined as *B. ermanii*. We have revised the sentence to clarify that in L317-318.

Finally, the results are not clear enough to draw any firm conclusions from the study, which casts doubt on the writers' expertise in the field.

**REPLY**: As your comments, our conclusions were vague in the manuscript of previous version. Then, we have added new section “Conclusions” to clarify the conclusions and what we found in this study.

**Responds to Reviewer #2**:

We appreciate your comments on every part of the manuscript. These significantly improved the manuscript. We attended to all suggestions like below.

However, the introduction could be strengthened by providing more context on why Betula ermanii was chosen as the focus of the study and why investigating ploidy levels in this species is important

**REPLY**: As your comment, we have added sentences providing our insights why *B. ermanii* was chosen and why ploidy levels of this species should be investigated in L76-79.

However, it would be helpful to include more information about the specific protocols followed for DNA extraction and flow cytometry analysis to ensure transparency and reproducibility.

**REPLY**: Thank you for the comment. As your comment, we added more specific explanation about fluorescence analysis in L166-169. Actually, we did not extract DNA but nuclei. Then, we revised the wording to clarify it in L162.

With some enhancements to depth and integration, the discussion could further elucidate the ecological and evolutionary significance of the observed patterns and provide more concrete recommendations for future research and conservation efforts.

**REPLY**: As your comments, our discussion in the manuscript of previous version needs enhancement of what we found. Then, we have added new section “Conclusions” to clarify the conclusions and what we found in this study.
